# Scientific perspectivism in the phenomenological tradition

**DOI:** 10.1007/s13194-020-00294-w

**Published:** 2020-06-16

**Authors:** Philipp Berghofer

**Affiliations:** Department for Philosophy, University of Graz, Heinrichstraße 26/5, 8010, Graz, Austria

**Keywords:** Scientific realism, Perspectivism, Phenomenology, Husserl, Merleau-Ponty, QBism

## Abstract

In current debates, many philosophers of science have sympathies for the project of introducing a new approach to the scientific realism debate that forges a middle way between traditional forms of scientific realism and anti-realism. One promising approach is perspectivism. Although different proponents of perspectivism differ in their respective characterizations of perspectivism, the common idea is that scientific knowledge is necessarily partial and incomplete. Perspectivism is a new position in current debates but it does have its forerunners. Figures that are typically mentioned in this context include Dewey, Feyerabend, Leibniz, Kant, Kuhn, and Putnam. Interestingly, to my knowledge, there exists no work that discusses similarities to the phenomenological tradition. This is surprising because here one can find systematically similar ideas and even a very similar terminology. It is startling because early modern physics was noticeably influenced by phenomenological ideas. And it is unfortunate because the analysis of perspectival approaches in the phenomenological tradition can help us to achieve a more nuanced understanding of different forms of perspectivism. The main objective of this paper is to show that in the phenomenological tradition one finds a well-elaborated philosophy of science that shares important similarities with current versions of perspectivism. Engaging with the phenomenological tradition is also of systematic value since it helps us to gain a better understanding of the distinctive claims of perspectivism and to distinguish various grades of perspectivism.

## Perspectivism in current debates

Advocating scientific realism, broadly speaking, means adopting “a positive epistemic attitude toward the content of our best theories and models, recommending belief in both observable and unobservable aspects of the world described by the sciences” (Chakravartty 2017). It is safe to say that scientific realism is the dominant view among the public. This is particularly due to the undeniable success of the sciences in making predictions and in contributing to the technological advancements we witness on a daily basis. Also among philosophers of science, the main motivation for adopting a realist position is the notorious miracle argument which quotes scientific realism as the best explanation for the obvious success of our scientific theories. This success would seem miraculous if our successful theories were misleading. Despite their initial plausibility, both scientific realism and the miracle argument have been attacked on many fronts. Objections based on the underdetermination of scientific theories by empirical data and the pessimistic meta induction gained such prominence in the twentieth century that Arthur Fine was prompted to declare: “Realism is dead” (Fine 1984, 83).

Currently, realism is again the dominant stance, but most realists concede that the attacks from the anti-realist camp require clarification and some sort of constraint of realist commitments. The most common response to the problems surrounding realism is to adopt a form of selective realism. Here entity realism and structural realism are the most prominent examples, but each version of realism suffers from certain shortcomings and by now scientific realism has splintered into a variety of different positions each being vigorously attacked not only by anti-realists but also by other realists. Accordingly, prominent voices have pointed out that the debate between realists and anti-realists has come to a “stalemate” (cf., e.g., Chakravartty 2018, 233; Forbes 2017, 3327; Frost-Arnold 2010, 56).

So where does this leave us? In recent debates, a new version of realism has emerged that is distinct from traditional versions of realism as well as from new versions of selective realism. This is *perspectival realism* or *scientific perspectivism*, in short, *perspectivism*. Its focus is not on certain parts of scientific theories (as it is the case for selective realism) but it aims at rethinking the nature and scope of scientific theories and models. The main works promoting perspectivism are Giere 2006, Massimi 2012, 2018a, b, and Teller 2001, 2011. Proponents of perspectivism typically view their position as a via media between objectivist realism and all forms of anti-realism. However, there is no unified picture of perspectivism; different proponents of perspectivism differ in their respective accounts. Here I will focus on the depiction of perspectivism offered in Giere 2006 since it seems to be closest to the ideas developed in the phenomenological tradition.

Giere contrasts his perspectival realism with what he calls objectivist realism:

I will be arguing that there is a kind of realism that applies to scientific claims that is more limited than this full-blown objective realism. Thus, in the end, I wish to reject objective realism but still maintain a kind of realism, a perspectival realism, which I think better characterizes realism in science. For a perspectival realist, the strongest claims a scientist can legitimately make are of a qualified, conditional form: ‘According to this highly confirmed theory (or reliable instrument), the world seems to be roughly such and such.’ There is no way legitimately to take the further objectivist step and declare unconditionally: ‘This theory (or instrument) provides us with a complete and literally correct picture of the world itself.’ (Giere 2006, 5f.)

Here we find three claims about scientific theories that Giere’s perspectivism subscribes to and that he takes to be in opposition to objective realism.

- P1: Scientific theories are fallible.
- P2: Scientific theories cannot be interpreted literally in their entirety.
- P3: Scientific theories cannot provide a complete picture of the world.

However, P1 and P2 are well-accepted claims and do not constitute a distinctive version of scientific realism. P1 is a claim *any* plausible form of scientific realism must accept. P2, as we have seen above, is advocated by all proponents of selective realism. Accordingly, what is distinctive about perspectivism should be encapsulated by P3. It is to be noted that P3 is an interesting and controversial claim that has been explicitly denied by prominent voices.^1^ Wilfrid Sellars expresses a widespread view within analytic philosophy of science when he says:

But, *speaking as a philosopher*, I am quite prepared to say that the common sense world of physical objects in Space and Time is unreal-that is, that there are no such things. Or, to put it less paradoxically, that in the dimension of describing and explaining the world, science is the measure of all things, of what is that it is, and of what is not that it is not. (Sellars 1963, 173)

As we will see below, this attitude that the common sense world is mere illusion and that science is *the* measure of reality, providing an exhaustive picture of all there is, is the main target of Husserl’s phenomenological philosophy of science. Sellars’ claim is also clearly opposed to P3. Let us take a closer look at how Giere motivates P3.

Giere focuses on three sources of human knowledge, arguing that all three of them only deliver perspectival knowledge. These three sources are color vision, scientific observation, and scientific theories. Concerning color vision, Giere summarizes his line of reasoning as follows:

For my purposes, maybe the most important feature of perspectives is that they are always partial. When looking out at a scene, a typical human trichromat is visually affected by only a narrow range of all the electromagnetic radiation available. In particular, the nearby wavelengths in the ultraviolet and infrared are simply not visually detected. And of course there is no possibility of visually detecting gamma rays or neutrinos. (Giere 2006, 35)

Giere is obviously right in stating that human color vision is perspectival in the sense of being partial and incomplete. What we call light, i.e., the visible spectrum, is only a very limited portion of the electromagnetic spectrum. The question is whether this is a *philosophically interesting* fact that suggests preferring perspectival realism over objectivist realism. Before we turn to this question in more detail, let us have a look at Giere’s further arguments based on his accounts of scientific observation and scientific theories.

His argument for the genuinely perspectival character of scientific observation resembles his argument concerning color vision.

Humans and various other electromagnetic detectors respond differently to different electromagnetic spectra. Moreover, humans and various other electromagnetic detectors may face the same spectrum of electromagnetic radiation and yet have different responses to it. In all cases, the response of any particular detector, including a human, is a function of both the character of the particular electromagnetic spectrum encountered and the character of the detector. Each detector views the electromagnetic world from its own perspective. Every observation is perspectival in this sense. (Giere 2006, 48)

For Giere, scientific observation is observation via instruments. But scientific instruments are structurally similar to human color vision in that human eyes as well as scientific instruments only engage with a limited scope of the electromagnetic spectrum. How we perceive the world, what we observe, and what data we gain crucially depends on the make-up of the respective methods we use to observe. Giere illustrates this by discussing how different types of telescopes produce very different images of the Milky Way (Giere 2006, 45–49).

The problem with Giere’s line of reasoning, as discussed so far, is that when we look at the examples he discusses, it remains unclear why there should be a clash with standard or objectivist scientific realism. What is the distinctive claim of perspectivism, embodied by the examples Giere provides, that standard forms of scientific realism must reject? Objectivist realists can clearly accept the fact that human vision and scientific observation only reveal limited aspects of nature. What they would insist on, however, is that the physical objects in question objectively and truly have the features ascribed to them. So when we say that from perspective X the object O has the feature F1 and from perspective Y the same object O has the feature F2, but F1 and F2 are not inconsistent, then there is no problem for objectivist realism.

In this context, Chakravartty indicates that in order to be interesting and at odds with realism, perspectivism must amount to “one or another form of relativism” (Chakravartty 2010, 406). However, this objection is problematic since Giere explicitly rejects “the unanalyzed assumption that a robust scientific realism must be objectivist realism because otherwise it slides into constructivism or relativism” (Giere 2006, 92). The problem is that Giere is not very precise in specifying the distinctive feature of perspectival realism that makes his form of realism non-objectivist but also non-relativist.

Chakravartty aims at clarification by making the following distinction:

As a philosophically controversial thesis, then, perspectivism would seem to take the form of either one or possibly both of the following claims: (In the sciences, in connection with representations such as theories and models, ...)

P1. We have knowledge of perspectival facts only, because non-perspectival facts are beyond our epistemic grasp.

P2. We have knowledge of perspectival facts only, because there are no non-perspectival facts to be known. (Chakravartty 2010, 407)

Chakravartty argues that none of the arguments and examples Giere provides support either of these strong theses. What is more, Chakravartty claims that there are simple counter-examples to P1 and P2. Such counter-examples are any facts that are intrinsically non-perspectival and can be known via scientific observation. “It is a non-perspectival fact about charged bodies, for example, that they exert electrostatic forces on other charged bodies” (Chakravartty 2010, 407).

It is hard to tell how Giere would respond to such examples. This is because Giere does not talk in terms of facts. Terms such as “scientific fact” or “perspectival fact” do not occur in Giere 2006. Instead, he stresses that scientific knowledge is always incomplete. With respect to naked-eye observation and scientific observation, he argues that they are always partial in the sense that they only capture certain aspects of the observed object as they appear from a certain perspective. This is a plausible claim. Importantly, this claim is not at odds with the claim that there are objective facts and that we can have knowledge of these facts. From my perspective, it appears that there is a laptop on my desk, this visual experience justifies me in believing that there is laptop on my desk, and it is objectively true that there is a laptop on my desk. We can think of similar examples regarding scientific observations. Accordingly, Giere’s claim that observation is perspectival and never provides a complete picture of the observed is not at odds with realism.

However, we can see how Giere’s perspectivism might be at odds with *objectivist* realism. By objectivist realism, apparently, Giere understands the view that the sciences deliver a *complete* picture of the world that is *free from all subjective factors*. It is not only the case that the world really has the features ascribed to it by science, there is nothing to know about the world over and above what is described by our best (possible) scientific theories. This means that when Giere discusses the perspectival character of scientific theories (Giere 2006, chapter 4), he argues that scientific theories are systematically similar to color vision and scientific observation. Unfortunately, Giere’s discussion of scientific theories is a bit messy. He captures the basic claim “that theoretical claims are also perspectival” in many different ways, suggesting many nonequivalent formulations and theses. Although he provides a general picture, the individual theses are not fleshed out in much detail. It would go beyond the scope of the present paper to discuss these controversial theses in detail. In what follows in this section, I will list some of these theses that I consider particularly interesting. In what follows in this paper, I will show what role these claims play in the phenomenological tradition.

(T1) Science itself is only a certain perspective we can have on the world. To describe or explain certain phenomena by virtue of a scientific theory, means to adopt a scientific perspective.^2^

(T2) Science and scientific theories can never deliver an exhaustive account of the world.^3^

(T3) If two scientific theories are inconsistent with each other, it does not follow that at least one of them is false. They might both shed light on different aspects of nature (cf. Giere 2006, 62, 94).

(T4) There is not and there cannot be one all-encompassing scientific theory to which all the other scientific theories can be reduced.

(T5) Science cannot be totally detached from the scientist who is doing science.^4^

In the following section, I will shed light on Husserl’s conception of horizontal intentionality, highlighting systematic similarities to Giere’s account of the perspectival character of observation. In Sect. 3, I illustrate that the claims T1, T2, and T5 play important roles in the phenomenological tradition. In Sect. 4, I discuss Merleau-Ponty’s partial realism that can be considered a radicalization of T5.

## Horizontal intentionality

As elaborated in the previous section, many philosophers of science have sympathies for the project of introducing a new approach to the scientific realism debate that forges a middle way between traditional forms of scientific realism and anti-realism. One such approach is perspectivism. Although different proponents of perspectivism differ in their respective characterizations of perspectivism, the common idea is that scientific knowledge is necessarily partial and incomplete. This *epistemological* claim can be supplemented by the stronger *ontological* thesis that the world itself is perspectival in the sense that at least some scientific facts are perspectival. Also, one can supplement it with the strong *methodological* claim that science must become aware of the fact that it is an agent-based endeavor in that it incorporates the first-person perspective into science. *Science must incorporate the scientist into science*.^5^

Perspectivism is a new position in recent debates but surely it has its forerunners. Massimi, for instance, points out that perspectivism “has a distinguished philosophical pedigree back to Leibniz, Kant, Nietzsche, and even Wittgenstein” (Massimi 2012, 25; cf. also Teller 2020). Matthew Brown argues that similar ideas can be found in Feyerabend and Dewey (Brown 2009; cf. also Giere 2016). Obviously, there are important similarities to ideas we find in Kuhn (Giere 2006, 2013) and in Putnam (De Caro 2020; Massimi 2018b).^6^ Interestingly, to my knowledge, there exists no work that discusses similarities to the phenomenological tradition.^7^ This is surprising because here one can find systematically similar ideas and even a very similar terminology. It is startling because early modern physics was noticeably influenced by phenomenological ideas.^8^ And it is unfortunate because the analysis of perspectival approaches in the phenomenological tradition can help us to get a more nuanced understanding of different forms of perspectivism. The main objective of this paper is historical: to show that in the phenomenological tradition one finds a well-elaborated philosophy of science that shares important similarities with current versions of perspectivism. However, there is also a systematic value in shedding light on the philosophy of science in the phenomenological tradition because it helps us to gain a better understanding of the distinctive claims of perspectivism and to distinguish various grades of perspectivism. Since it is one of the main problems of current perspectivism to make clear how exactly it differs from standard forms of realism or anti-realism, the present elaboration can contribute to a better understanding of the distinctiveness of perspectivism. We will start with claims shared by most phenomenologists (Sect. 3) and see how these ideas can be radicalized (Sect. 4).

This section discusses Husserl’s conception of horizontal intentionality, clarifying to what degree it is in agreement with Giere’s analysis of the partial nature of observation. We will also get a first glimpse of the phenomenological doctrine that there is no view from nowhere available, not even to science.

One of Husserl’s main contributions to a proper phenomenological analysis of perceptual experience is his disclosure of what he calls the horizontal structure of experience. Perceptual experiences go beyond what is directly given.^9^ When you look at the laptop in front of you, your experience presents to you the laptop’s screen, case, keyboard, etc. Your experience has a presentive or in Husserl’s terminology a self-giving character with respect to these aspects of your laptop. But your perceptual experience does not only intend these directly perceived aspects. What is *co-given* to you *within experience* is the laptop’s back, that its back has certain properties such as a smooth surface, that it also has a smooth underside, etc. You do not actually see the back or underside of your laptop but they are co-intended aspects of your experience.

Furthermore, the laptop is not experienced as an isolated object but as embedded in a surrounding world. Even if your attention is solely directed at the laptop, it is part of your experience that the laptop does not float around in nothingness, but is placed on a desk, which in turn stands on the floor here in your office at the department of your university in your hometown and so on. In Husserl’s terminology, these co-given objects (desk, floor, etc.) are part of the *outer horizon* of your experience. The hidden but co-intended aspects of the object itself (the laptop) are part of the *inner horizon* of your experience (Husserl 1972, 28). Husserl characterizes the perspectival character of perception as follows:

Of necessity a physical thing can be given only ‘one-sidedly;’ […] A physical thing is necessarily given in mere ‘modes of appearance’ in which necessarily a *core of* ‘*what is actually presented*’ is apprehended as being surrounded by a horizon of ‘*co-givenness*,’ which is not givenness proper. (Husserl 1982, 94).

This means that perception always “implies a *plus ultra*” (Husserl 2001, 48). According to Husserl, the perspectival character and horizontal structure of perception is not simply a result of the imperfection of human beings but an essential property of perception. Not even a god could change that perceptual experiences present their physical objects always in perspectives (Husserl 1982, 95).

When Husserl illuminates the perspectival character of perception, he not only stresses that perception is incomplete but also that physical objects in perception always appear from a certain point of view.

All orientation is thereby related to a null-point of orientation, or a null-thing, a function which my own body has, the body of the perceiver. And again, the perspectival mode of givenness of every perceptual thing and of each of its perceptual determinations – on the other hand, also of the entire unitary field of perception, that of the total spatial perception – is something new. The differences of perspective clearly are inseparably connected with the subjective differences of orientation and of the modes of givenness in sides.^10^ (Husserl 1977, 121)

A further aspect of perception is that previous experiences shape the way we perceive. Perception is not a faculty that allows us to see the world as it is objectively, independent from our history, background beliefs, etc. To put it differently, “experience is not an opening through which a world, existing prior to all experience, shines into a room of consciousness; it is not a mere taking of something alien to consciousness into consciousness” (Husserl 1969, 232). This aspect of perception is closely related to discussions about the theory-ladenness of perception.

Obviously, there are many similarities between Husserl’s conception of horizontal intentionality and Giere’s analysis of perception. For Giere, human perception is always incomplete because we only engage with a restricted part of the electromagnetic spectrum. For Husserl, perception is essentially incomplete because it presents its objects only one-sidedly and from a specific perspective. What is more, perception is shaped by subjective factors such as one’s history, culture, or previous experiences. It should be noted, however, that there are also crucial differences between Husserl and Giere. Husserl aims at making a general point about perceptual experiences based on descriptive phenomenological reflection. For Husserl, it is essentially (a priori) true that the object of a perceptual experience, i.e., a physical object extended in space, can only be given perspectivally. Giere’s reasoning, on the other hand, is based on results of the empirical sciences, particularly on how human eyes interact with the electromagnetic spectrum. Notwithstanding the differences in scope and methodology, this might be a good example where phenomenological and empirical investigations come to similar conclusions, thereby complementing each other.

Although perceptual experiences are necessarily perspectival and also shaped by previous experiences and further subjective factors, for Husserl this does not mean that perception cannot be a source of epistemic justification. In fact, for Husserl, perceptual experiences are the prime examples of sources of justification. According to Husserlian phenomenology, epistemology is intrinsically linked to the study of the intentional structures of consciousness. The most fundamental epistemological question turns out to be how subjectivity can be the source of knowledge (cf. Melle in Husserl 1984, page XXXI). Subjectivity is not only at the center of epistemology because justification is always justification for a subject. More importantly, justification is gained through subjective acts: “Subjective acts motivate everything” (Husserl 1984, 121). Here “subjective acts” simply means intentionally directed mental states of a subject. But which acts are justification-conferring? The answer is experiences. More precisely, those mental states that have a presentive character, that present their objects in an intuitive (“anschaulich”) manner. For Husserl, this not only includes perceptual experiences but also, e.g., introspective intuitions and a priori intuitions (for more details on Husserl’s conception of experiential justification, cf. Berghofer 2018a, 2020). As we have seen above, although Husserl regards experiences as a source of immediate justification, he is well aware that experiences are not windows to the world through which we see how the world is in itself thoroughly objectively. Instead, experiences present their objects in a certain way that at least partly depends on subjective factors such as previous experiences, background beliefs, etc. In this context, Husserl emphasizes the role of transcendental subjectivity.

Every imaginable sense, every imaginable being, whether the latter is called immanent or transcendent, falls within the domain of transcendental subjectivity, as the subjectivity that constitutes sense and being. The attempt to conceive the universe of true being as something lying outside the universe of possible consciousness, possible knowledge, possible evidence, the two being related to one another merely externally by a rigid law, is nonsensical. They belong together essentially; and, as belonging together essentially, they are also concretely one, one in the only absolute concretion: transcendental subjectivity. If transcendental subjectivity is the universe of possible sense, then an outside is precisely – nonsense. (Husserl 1960, 84)

Passages like this have led to much controversy in Husserl scholarship, some interpreting this as a methodological claim (as I do), others interpreting it as a commitment to metaphysical idealism. For our purpose, it suffices to note that for Husserl a view from nowhere at the world is in principle impossible. This is true not only for our perceptual encounter with the world but also for our scientific encounter.

## The scientific perspective

Husserl’s main work concerning philosophy of science is his *The Crisis of European Sciences and Transcendental Phenomenology*. According to Husserl, the success of modern science (beginning with Galileo), i.e., the success of mathematizing and quantizing nature, has led to several misunderstandings. Firstly, it made scientists, as well as philosophers, believe that the methods of science are the only legitimate forms of gaining knowledge. Husserl opposes this methodological naturalism that implies that even philosophy must proceed like a natural science. Secondly, Husserl bemoans that due to the objectivism of modern age it has become commonplace to take the mathematical models and formulae to be the true reality, while the life-world, the world of our everyday experiences, the world of tables and chairs, is demoted to some kind of illusion (cf. particularly Husserl 1970, 48–53).^11^ Husserl emphasizes that mathematics and geometry are only *methods* to describe physical reality, they are not the “true” reality lying behind what we can intuitively (“anschaulich”) observe. What he criticizes is that scientists and philosophers seem to have forgotten this and tend to confuse what is a method with what is reality. He stresses that the life-world serves as the epistemic grounding^12^ and the meaning-foundation of all scientific activities.^13^

Husserl’s conception of the life-world proved useful in many contexts and his thesis that the life-world remains the epistemic foundation for any scientific theory should be particularly interesting to proponents of perspectivism.^14^ However, if you wish to abstain from using the terminology of a life-world, the basic idea remains that no matter how abstract your scientific theories are, their justification, ultimately, lies in ordinary experiences, in what is immediately given. In Husserl’s words, “the inductive scientific judging” of the “exact objective sciences that by going beyond the immediately experienced deduces the non-experienced is always dependent on its ultimate legitimizing basis, on the immediate data of experience” (Husserl 1973, 121; my translation).

To be sure, Husserl neither criticizes science per se nor its methods. And, of course, he does not dispute its success. The sciences, particularly physics, are extremely successful in what they are doing. However, he criticizes the conclusions naturalists and objectivist realists draw from the success of science. According to Husserl, the scientific method is not the only method of gaining knowledge, science does not provide an *exhaustive* picture of nature, and science is not completely independent from subjective factors.

Edith Stein, one of Husserl’s foremost pupils, put it this way: “What physics [...] reveals pertains to the real nature but it never *exhausts* nature. And what evades the web of mathematical formulas is not less ‘real’ than what is captured by mathematics” (Stein 2004, 62). Here we find three motifs that are typical for a phenomenology of physics. First, phenomenology does not dispute the success of physics, neither does it object to the implementation of mathematics. Secondly, however, the mathematical picture delivered by physics only reveals one perspective of nature.^15^ Even if we manage to get a mathematical grip on nature, what we gain from this can never be an exhaustive picture of nature. “We have seen that the methods of the exact natural sciences do not capture reality in its totality, instead they are only concerned with certain sides of nature” (Stein 2004, 73).^16^ Thirdly, not being mathematizable does not imply not being real. This is not only true for certain aspects of nature but also for totally different entities including values, essences, and consciousness. Mathematics is extremely useful in physics but this does not imply that any science (including philosophy, value theory, etc.) must attempt to mathematize its objects.

The idea that scientific knowledge is perspectival and dependent upon the scientist’s experiences and life-world features also prominently in the work of Merleau-Ponty. “Everything that I know about the world, even through science, I know from a perspective that is my own or from an experience of the world without which scientific symbols would be meaningless. The entire universe of science is constructed upon the lived world” (Merleau-Ponty 2012, lxxii).

Importantly, such phenomenological reflections on the basic epistemic role of the life-world are supported by recent conceptions of agent-based modeling (Giere 2010) and by experimental results concerning the relationship between perceptual and conceptual learning (Landy & Goldstone 2007). The upshot is as follows: All we know about the physical world, we know, ultimately, by way of perceptual experiences. But our experiences do not constitute some magical purely objective view from nowhere. Our experiences are shaped by previous experiences as well as by our beliefs and expectations. What is more, perceptual experiences affect our concept formation and symbolic thinking, and the concepts we use, in turn, affect our experiences. Accordingly, when we do physics, when we establish a mathematical model that is intentionally directed at the world, we do not have a purely objective mathematical model on the one hand and objective reality on the other hand. Instead, on both sides we have models influenced by subjective factors.

Referring to a famous example put forward by Arthur Eddington (Eddington 1928, ixf.), I may exemplify the above as follows: Looking at this table in front of me is a perceptual act (precisely but perspectivally) directed at this table in front of me. A physical-mathematical model of this table, describing its atomic structure etc., is also (precisely but perspectivally) directed at this table. Both are legitimate ways of being intentionally directed at the table, each elucidating *different* aspects of the *same* object.^17^ They complement each other and in certain ways they influence each other. They are both legitimate perspectives, one of them being epistemologically more fundamental. It is our everyday experience that is epistemologically more fundamental because this is the one from which our mathematical model arises and the one the mathematical model, ultimately, must conform to.^18^

We know from Mirja Hartimo’s analysis of Husserl’s private library that Husserl was not only interested in modern physics but that he studied it in great detail (Hartimo 2018). However, Husserl never practiced philosophy of physics in a narrow sense. He never engaged with, say general relativity, to draw philosophical conclusions. And he never attempted a phenomenological interpretation or grounding of general relativity or quantum mechanics.^19^ This is a bit surprising since many physicists, as well as many philosophers of physics, believe that the downfall of classical mechanics and the rise of general relativity and particularly quantum mechanics support key ideas of Husserl’s phenomenology of science.^20^

Concerning general relativity, Merleau-Ponty states:

The physics of relativity confirms that absolute and final objectivity is a mere dream by showing how each particular observation is strictly linked to the location of the observer and cannot be abstracted from this particular situation; it also rejects the notion of an absolute observer. We can no longer flatter ourselves with the idea that, in science, the exercise of a pure and unsituated intellect can allow us to gain access to an object free of all human traces, just as God would see it. This does not make the need for scientific research any less pressing; in fact, the only thing under attack is the dogmatism of a science that thinks itself capable of absolute and complete knowledge. We are simply doing justice to each of the variety of elements in human experience and, in particular, to sensory perception. (Merleau-Ponty 2004, 44f.)

It is to be noted that Merleau-Ponty’s remark is misleading since in the theory of relativity observation is not linked to the location of the observer but to the *frame of reference* of the observer.^21^ The principle of relativity implies that there is no privileged frame of reference; the laws of physics are the same in all inertial frames of reference. Special relativity is built upon the principle of relativity (first postulate) and the postulate that in a vacuum the speed of light is constant for all observers. Together, these two postulates have several implications that show that some of the facts that we usually consider to be “objective” are in fact observer-dependent. For instance, special relativity implies the relativity of simultaneity: It depends on the observer’s frame of reference whether two events separated in space occur at the same time. There is no objective or absolute sense in which we could tell that two spatially separate events take place simultaneously. When we turn to *general* relativity, we see that space and time are not absolute, not a fixed background, but that the geometry of spacetime itself is influenced by what is going on within spacetime, namely by the energy-momentum of matter. This means that there is a reciprocal relationship between spacetime and what it contains (including the embodied observer).^22^

All this deserves phenomenological reflections on its own, but the theory of relativity is no focus of this paper. Instead, in the following two sections, we will address how perspectivist and phenomenological approaches to science relate to *quantum mechanics*. This focus on quantum mechanics arises naturally because when Merleau-Ponty spells out his phenomenological perspectivism, he crucially draws on quantum mechanics. Among the most famous of the classical phenomenologists (Husserl, Scheler, Heidegger, Stein, Sartre, Merleau-Ponty), Merleau-Ponty was the one most explicitly engaging with the natural sciences. He believed that modern physics supports phenomenological approaches to science and reality and although he also engaged with the theory of relativity, he believed that quantum mechanics best supports phenomenological approaches. In the following section, we shed light on Merleau-Ponty’s phenomenology of physics, emphasizing how he radicalizes ideas we find in Husserl. In the final section, we shall see that there are interesting systematic similarities between ideas we find in Merleau-Ponty and a current popular interpretation of quantum mechanics.

## Merleau-Ponty’s partial realism

Merleau-Ponty has a reputation for having deeply cared about the natural sciences, particularly psychology. This makes him a promising and popular point of origin for many contemporary phenomenologists working on the interface to psychology and the cognitive sciences. It is less well known, however, that he also explicitly addressed physics, contemplating how philosophy and physics can enrich each other and what a phenomenologically grounded physics may look like. This is particularly true for his “Modern Science and Nature” which is part of the lecture courses published in *La Nature*. Here, Merleau-Ponty carefully engages with quantum mechanics, outlining his phenomenological approach to physics.

Merleau-Ponty discusses the limits of objectivity and aims at a physics that takes into consideration the physicist who observes and experiments. He believes that modern physics, particularly quantum mechanics, exemplifies or at least leads to a new kind of science that engages in self-criticism, reflectively addressing its relationship to the objects it studies (Merleau-Ponty 2003, 85). In this context, he discusses and draws on the interpretation of measurement in quantum mechanics delivered by London and Bauer (London & Bauer 1939; 1983) that was itself heavily influenced by Husserl’s phenomenology.^23^

For Merleau-Ponty, physics in its most perfected form abandons the idea of delivering a completely objective picture of the world. Instead, physics needs to put the physicist into physics and account for the fact that the life-world predates all scientific endeavors. In his words:

But a physics that has learned to situate the physicist physically, a psychology that has learned to situate the psychologist in the socio-historical world, have lost the illusion of the absolute view from above: they do not only tolerate, they enjoin a radical examination of our belongingness to the world before all science. (Merleau-Ponty 1968, 27)

There is an increasing number of philosophers and physicists who insist “that immediate experience and the world can never be separated” and agree that science cannot “give us a complete, objective description of cosmic history, distinct from us and our perception of it.”^24^ Doubting “that the physical object in itself” pre-exists physics, Merleau-Ponty considers the “relations between the observer and the observed” to be the “ultimate physical beings” (Merleau-Ponty 1968, 15). Underpinned by the London & Bauer account of quantum mechanics, he doubts “the idea that every object has an individual existence,” and instead refers to physical objects as “generic realities” (Merleau-Ponty 2003, 92).

Furthermore, he explicitly addresses one of the most important questions of philosophy of science: What do we observe in scientific measurements? Of course, this question is particularly pressing in quantum mechanics. Contrasting the role of the measuring apparatus in classical physics and quantum mechanics, Merleau-Ponty states that while classically “the apparatus is the prolongation of our senses” in quantum mechanics “[t]he apparatus does not present the object to us.” Instead, “[i]t realizes a sampling of this phenomenon as well as a fixation. […] Known nature is artificial nature” (Merleau-Ponty 2003, 93).

Unfortunately, Merleau-Ponty does not offer a detailed and sophisticated analysis of what is “artificial” about quantum measurements. In general, in his discussion of quantum mechanics we find much that is inspiring and helpful but his remarks are often vague and in order to advance current debates we would need a more precise analysis of how quantum mechanics supports perspectivist and phenomenological approaches. The next section is intended to make a step towards this goal. This being said, I believe that Merleau-Ponty is right and that there is a fundamental difference between looking through telescopes or microscopes on the one hand and using measuring devices in quantum mechanics on the other hand. In the case of telescopes and microscopes, there is a rather straightforward sense in which we directly observe the object in question. But it would be quite a stretch to say that the same is true when looking at the *photographs* gained by cloud chambers and bubble chambers that visualize the *tracks* of charged particles.^25^ What is more, while cloud chambers and bubble chambers have a photographic readout, the devices that are now common, such as particle colliders like the LHC, have a purely electronic readout. What we gain from LHC experiments is data – big data. “Data pours out of the LHC detectors at a blistering rate. Even after filtering out 99% of it, in 2018 we gathered 88 petabytes of data.”^26^ I do not say that there is anything genuinely problematic about this process but it is a process that needs careful philosophical-phenomenological reflection and it is a process that is far from delivering a purely objective picture of the world.^27^

The point is that similar to Giere, Merleau-Ponty rejects the idea that scientific observation provides us with an objective picture of nature. Of course, they differ in their respective reasons for rejecting this idea. Giere emphasizes that scientific instruments only engage with a limited aspect of nature. Merleau-Ponty seems to stress that according to quantum mechanics the act of observing inevitably affects the observed reality (e.g., when electrons, depending on the experimental setup, either behave like particles or like waves).

Another important similarity to Giere is that Merleau-Ponty aims at establishing a position that is in between objectivist realism and anti-realism, namely a version of realism that rejects the ideal of providing a complete and purely objective view of the world and that takes into account the subject that is doing science. To get a better grip on Merleau-Ponty’s approach to the scientific realism debate, let us see which positions he rejects. One might think that phenomenologists feel sympathetic to instrumentalist accounts (and for some phenomenologists this is certainly true), but Merleau-Ponty clearly opposes such views. Merleau-Ponty does not use the term “instrumentalism,” but he introduces the following position:

Physics should not be conceived as a search for the truth, it should give up determining a real physics: it would be only an ensemble of measurements linked to equations, allowing [us] to foresee the result of future measurements. Formalist physics receives all freedom, but it loses its ontological content. It signifies no mode of being, no reality. (Merleau-Ponty 2003, 95f.)

This position, expressing the core ideas of instrumentalism, is rejected by him without much argument. For Merleau-Ponty, it is clear that physics, correctly interpreted, indeed tells us something significant about the nature of reality. Physics is not a mere tool to make predictions, nor can reality be reduced to what is measured and observed.

He goes on, drawing on the work of the French physicist and logician Paulette Destouches-Fevrier, to point out that it would also be a mistake to adopt an idealist position. The problem with idealism is that just like standard realism it amounts to a form of objectivism. To be more precise, idealism is an objectivism that “objectifies human representations” (Merleau-Ponty 2003, 96). Instead, Merleau-Ponty is convinced that “[t]he relations between reality and measurement must be conceived outside of the dichotomy of in-itself/representation” (Merleau-Ponty 2003, 96). Acknowledging that “[p]hysics cannot be realist in the classical sense” but “cannot be idealist, either,” Merleau-Ponty chooses to term his position “a ‘partial realism’ or a ‘participationist’ conception” (Merleau-Ponty 2003, 97f.). This terminology, adopted from Paulette Destouches-Fevrier, highlights the interrelatedness and inseparability of the observer and the observed. The term “partial realism” emphasizes that although this view is not a traditional form of realism, it is supposed to be some form of realism. Returning to our initial question of how to place Merleau-Ponty in the scientific realism debate, we need to ask: which form of realism? What are the fundamental objects of reality according to his partial realism, his participationist conception?

In this context, he calls reality a “structural plane,” and continues by quoting a long passage from Destouches-Fevrier.^28^ Here Destouches-Fevrier says that reality “transcends the opposition object-subject.” The focus is on the “structural relations” between subject and object. These structural relations “refer not to an object, but to certain mathematical forms necessary for the description of the relation of the subject to the object.” The ontological significance of the structural relations is highlighted by pointing out that “they are independent of the results of the processes of measurement” and by perhaps misleadingly comparing them “to the Platonic objectivity.” (Destouches-Fevrier quoted in Merleau-Ponty 2003, 98).

Unfortunately, Merleau-Ponty does not do much to clarify or go beyond these remarks of Destouches-Fevrier. However, in *The Visible and the Invisible* he holds that modern physics is obliged “to recognize as ultimate physical beings in full right relations between the observer and the observed” (Merleau-Ponty 1968, 15). In this light, there is little doubt that Merleau-Ponty subscribes to the structuralist view drafted by Destouches-Fevrier.

Accordingly, concerning our questions of how to understand the fundamental objects of reality according to Merleau-Ponty’s partial realism, we get the following answer: The fundamental objects are the structural relations between the observer and the observed. These relations can neither be reduced to the objective nor to the subjective. Reality transcends this opposition. Reality can only be understood or even consists in the relations between the observer and the observed.^29^ Admittedly, all this remains vague. However, in the next section, we shall see that there is a novel interpretation of quantum mechanics that agrees with Merleau-Ponty that quantum mechanics should be understood as revealing the fundamental relatedness between subject and object: QBism. Before turning to a more substantial discussion of quantum mechanics, let me briefly recapitulate and put into perspective what we have achieved so far.

In Sect. 1, we have seen that for Giere science is an agent-based endeavor. This idea is fleshed out by Husserl in terms of the life-world serving as the meaning-foundation for all the individual sciences. Merleau-Ponty radicalizes this idea by making the ontological claim that reality is in some sense observer-dependent (perhaps not necessarily mind-dependent). Of course, phenomenologists are not committed to this strong claim of observer-dependence, ontologically understood. Husserl argues that subjectivity and subjective experiences constitute the source of all knowledge and justification but this is an epistemological claim and Husserl’s transcendental idealism can be interpreted as a methodological-epistemological project free from strong metaphysical implications. According to Husserl’s understanding of the mathematical sciences including theoretical physics, these sciences strive for a maximum of objectivity by looking at the world from the third-person perspective, mathematizing and quantizing their target system. And although full objectivity can never be gained because the scientists’ life-worlds remain the epistemic grounding and meaning-foundations for all scientific theories, for Husserl there is nothing wrong with the individual sciences to proceed in this manner.^30^ We only need to be careful in how to interpret scientific theories and be aware of their limitations.^31^

Merleau-Ponty goes beyond such interpretational claims. There is at least one methodological and one ontological claim he adds.^32^ The *methodological* claim is that the sciences, particularly physics, must refrain from aiming at a purely objective account of the world. Instead, they must incorporate the first-person perspective into science. Only by doing so can science unveil the most fundamental structures of reality. The *ontological* claim is that reality is essentially observer-dependent and that the fundamental objects are relations between the observer and the observed. Merleau-Ponty’s position seems to qualify as a version of *perspectival realism* since he rejects objectivist realism and aims at a position in between objectivist realism and anti-realism. Scientific theories are perspectival in the sense that they need to incorporate the scientists’ relations to the objects. Scientific facts are perspectival in the sense that they depend on, or, perhaps more accurately, consist of the scientists’ relations to the objects.

Nevertheless, one might doubt that Merleau-Ponty’s partial realism is a form of realism after all. This is because scientific realism is usually associated with an ontological commitment to the mind-independence of reality. However, we have to note, first, that Merleau-Ponty’s partial realism does not amount to a form of traditional anti-realism (instrumentalism, idealism, constructivism) and, secondly and more importantly, that Merleau-Ponty would strongly deny the anti-realist claim that the sciences cannot tell us anything about the nature of reality. In this regard of aiming to be in between standard versions of realism and anti-realism, Merleau-Ponty’s partial realism shares significant similarities with QBism, classified by its chief advocate Christopher Fuchs as a “participatory realism.” Fuchs insists that quantum mechanics must be understood “as being part of a realist program, i.e., as an attempt to say something about what the world is like, how it is put together, and what’s the stuff of it” (Fuchs 2017, 117). Importantly, according to Fuchs, the conclusions he draws from quantum mechanics are not something we need to artificially read into it. Quite the contrary, “[q]uantum theory itself threw these considerations before us!” (Fuchs 2017, 115). This means we need to take quantum mechanics, its features and phenomena, at face value and by doing so we learn something new about reality that was hidden in classical physics.

I believe that this perfectly captures Merleau-Ponty’s attitude towards quantum mechanics. Quantum mechanics does not deliver a purely objective view on the world as the objectivist realist would have it; instead, quantum mechanics shows us that a purely objective view on the world is impossible. Quantum mechanics does not represent an observer-independent reality; instead, quantum mechanics calls into question the idea that there is a purely observer-independent reality behind the phenomena. How quantum mechanics might support perspectivist and phenomenological approaches to science and how QBism relates to Merleau-Ponty’s ideas are the topics of the following section.

## QBism

In quantum mechanics we find many concepts and phenomena that seem to support perspectivist and phenomenological approaches to science and reality, undermining our classical world-view. Determinism is called into question, Heisenberg’s uncertainty principle imposes certain limitations on what we can know about reality, and complementarity and entanglement are often viewed as revealing that acts of measurement necessarily affect observed reality. Particularly in the early days of quantum mechanics, complementarity was considered the key feature of quantum mechanics and was interpreted in a way that seems to support a perspectivist picture. Heisenberg summarized the Copenhagen understanding of complementarity as follows:

By this term ‘complementarity’ Bohr intended to characterize the fact that the same phenomenon can sometimes be described by very different, possibly even contradictory pictures, which are complementary in the sense that both pictures are necessary if the ‘quantum’ character of the phenomenon shall be made visible. (Heisenberg 1977, 6)

Wave-particle duality and Heisenberg’s uncertainty principle are often considered the most prominent manifestations of complementarity. Heisenberg’s uncertainty principle famously says that with respect to complementary variables such as position and momentum the more precisely we determine the one, the less we know about the other. As Heisenberg himself noted this implies that “[e]ven in principle we cannot know the present in all detail” (Heisenberg 1983, 83). Concerning measurements in quantum mechanics, Frescura and Hiley express a common attitude among physicists when they say that the issues surrounding complementarity imply

that not all aspects of a system can be viewed simultaneously. By using one particular piece of apparatus only certain features could be made manifest at the expense of others, while with a different piece of apparatus another complementary aspect could be made manifest in such a way that the original set became non-manifest, that is, the original attributes were no longer well defined (Frescura & Hiley 1984).

This claim that scientific instruments can only shed light on certain limited aspects of reality is precisely the claim we find in Giere (2006, chapter 3) as discussed in section 1. However, concerning the examples discussed by Giere, one might argue that one could simply add all the perspectives delivered by different instruments to gain one complete picture of reality. Complementarity in quantum mechanics, on the other hand, seems to impose even more rigorous limitations on scientific observations, revealing a genuinely perspectival element of science that disallows gaining one complete picture by adding different perspectives: An increase of information with respect to one set of properties goes hand in hand with a decrease of information with respect to another set of properties. In this picture, quantum mechanics reveals limits to objectivity in the sense that our knowledge of quantum systems is necessarily perspectival, we can always only know certain aspects, never nature in its entirety.

Of course, drawing such conclusions from quantum mechanics is a minefield since there is no consensus on its ontological as well as epistemological implications. Interpreting quantum mechanics, offering a solution to the notorious measurement problem, is often considered the main topic of philosophy of physics. As a consequence, there exist a number of different interpretations that lead to very different pictures of reality. This is true also for the question of which conclusions to draw from Heisenberg’s uncertainty principle (cf. Hilgevoord & Uffink 2016).^33^

Many open questions in quantum mechanics concern the wave function and its apparent collapse. Is the wave function something that really exists or is it merely a mathematical tool, useful to make predictions? Why is it that apparently the outcomes of measurements are always definite states? Does the wave function collapse upon measurement? If so, how and why? This is where so-called interpretations of quantum mechanics usually come into play. Some of them, most notably Bohmian mechanics and the many-worlds interpretation, preserve (in some sense and with some costs) the deterministic picture we know from classical mechanics. Proponents of these interpretations typically view the wave function as physically real and believe that quantum mechanics provides an objective picture of reality. Other interpretations, such as Rovelli’s relational interpretation (Rovelli 1996), Dieks’ perspectivalism (Dieks 2019a, 2019b), or Healey’s pragmatist approach (Healey 2012) in one way or another contest the idea that physics delivers a purely objective picture of the world. One interpretation in this camp that has gained particular attention recently is QBism. I shall focus on QBism also because the ideas and the terminology we find here are particularly close to what we found in Merleau-Ponty.

To be sure, I do *not* claim that perspectivists or phenomenologists must or should subscribe to QBism. Nor do I argue that they are committed to any “subjective” interpretation of quantum mechanics. However, QBism might be the interpretation that most consistently promotes some of the ideas we find in phenomenology, which is why it is worth considering it in more detail (cf. particularly Bitbol forthcoming and Tremblaye forthcoming).

In QBism the agent and her experiences and expectations play a central role. The distinctive idea of QBism is to apply a personalist Bayesian account of probability, as it has been developed by Bruno de Finetti, to quantum probabilities. This means that *probabilities in quantum mechanics* are interpreted not as objective but as *subjective probabilities*. Accordingly, in QBism *quantum states* do not represent objective reality but instead *represent an agent’s subjective degrees of beliefs about her future experiences*. Consequently, the wave function is not physically real but a mathematical tool that encodes one’s expectations about one’s future experiences. In short, QBism argues that quantum states do “not represent an element of physical reality but an agentʼs personal probability assignments, reflecting his subjective degrees of belief about the future content of his experience” (Fuchs & Schack 2015, 1).

The first thing to note is that even opponents of any subjective interpretation of quantum mechanics concede that such an account delivers a straightforward solution to the notorious apparent collapse of the wave function. This is because any “approach according to which the wave function is not something real, but represents a subjective information, explains the collapse at quantum measurement perfectly: it is just a process of updating the information the observer has” (Vaidman 2014, 17). To put it differently, according to QBism “[t]he notorious ‘collapse of the wave-function’ is nothing but the updating of an agent’s state assignment on the basis of her experience” (Fuchs et al. 2014, 749). In this sense, QBism dissolves the measurement problem – the problem does not even show up.^34^

A further advantage of QBism is that it avoids certain implausible consequences that plague realist interpretations of the wave function. Mathematically speaking, wave functions are vectors in a Hilbert space. This is often expressed by saying that “**Wave functions live in Hilbert space**” (Griffiths 2018, 94). A Hilbert space is an abstract mathematical concept, namely a complete vector space on which an inner product is defined. But if the wave function is something real, does this mean that mathematical Hilbert space is physically real too? In fact, one can find prominent voices championing Hilbert space realism (e.g. Carroll & Singh 2019) but most consider this an implausible and unwarranted mathematization of nature and it has been pointed out that only “[v]ery few people are willing to defend Hilbert space realism in print” (Wallace 2013, 216).

A similar but more subtle form of mathematization takes place in configuration space realism, i.e., the project of reifying the 3 *N*-dimensional configuration space, N being the number of the particles in the universe. The main proponent of this view is David Albert, who at one point considered our impression that we live in three-dimensional space “somehow flatly illusory” (Albert 1996, 277). Configuration space realism, often referred to as “wave function realism,” has been quite popular and has sparked much controversy. However, if configuration space realism is meant to imply that the physical space of our everyday experiences is demoted to some kind of illusion, then this position is in danger of being empirically incoherent.^35^ Furthermore, one might object that configuration space realism “makes the same unmotivated conceptual move as Hilbert space realism: it reifies a mathematical space without any particular justification” (Wallace 2013, 217).

Concerning the formal and technical apparatus of the mathematical sciences, Husserl warned us not to be “misled into taking these formulae and their formula-meaning for the true being of nature itself” (Husserl 1970, 44). We see that this problem also arises in quantum mechanics via wave function realism. The most common realist interpretations of quantum mechanics, the many-worlds interpretation, Bohmian mechanics, and GRW theory are all in danger of leading to a mathematization of nature that is not based on physical principles but on mathematical formalism. QBists such as Christopher Fuchs and Blake Stacey have pointed out that in these interpretations “the strategy has been to reify or objectify all the mathematical symbols of the theory and then explore whatever comes of the move” (Fuchs & Stacey 2019, 136).

QBism takes a different approach. Instead of reifying mathematical constructs, the idea is “to reduce the mathematical structure of quantum mechanics to some crisp physical statements” (Fuchs & Stacey 2016, 285). In this respect, QBism is similar to informational approaches to quantum mechanics that seek to reconstruct quantum mechanics based on fundamental physical principles (cf., e.g., Bub 2004, Chiribella et al. 2011, and Goyal 2012). A common idea is that “[i]n quantum mechanics, *maximal information is not complete and cannot be completed*” (Caves et al. 2002, 3) and according to QBists this result “*can be regarded as the greatest triumph of Bayesian reasoning*” (Caves et al. 2002, 3). I take it to be a virtue of QBism to resonate well with recent developments in quantum information theory and the insistence that information is necessarily incomplete also resonates well with perspectivism.

So far, we have seen that the key move of QBism is to interpret quantum probabilities as personalist Bayesian probabilities and that this move allows QBism to dissolve the measurement problem and to avoid implausible mathematizations of nature. But what about more substantive claims about how reality works? Does QBism amount to some sort of instrumentalism according to which quantum mechanics does not teach us anything about reality? Indeed, the charge of instrumentalism is one of the most common objections to QBism.

Importantly, proponents of QBism, particularly Christopher Fuchs, vehemently deny such anti-realist interpretations of QBism. Instead, Fuchs argues that, according to QBism, quantum mechanics *tells us something very important about reality*, namely “that reality is *more* than any third-person perspective can capture” (Fuchs 2017, 113). In this spirit, one of the objectives of QBism is to put the scientist back into science (Mermin 2014). Fuchs chose the label “participatory realism” for QBism due to the prominent role that the subject and her experiences play in QBism, highlighting the interrelatedness of subject and object (Fuchs 2017).

Interpreting probabilities in quantum mechanics as subjective probabilities is only the *starting point* of the QBist project. The idea is that the fact that quantum mechanics does not deliver a purely objective picture of reality is not a shortcoming of the theory, instead quantum mechanics tells us that reality does not allow being objectively captured at a fundamental level.

But what exactly does it mean when Fuchs calls QBism a “participatory realism”? How exactly are subject and object interrelated? Arguably, this concerns the most challenging aspect of QBism and it does not seem that a comprehensive philosophical foundation has been offered to address this question. However, to get a better idea of what QBists have in mind, we turn to the concept of measurement. According to QBism, “[a] measurement does not, as the term unfortunately suggests, reveal a pre-existing state of affairs. It is an action on the world by an agent that results in the *creation* of an outcome – a new experience for that agent” (Fuchs et al. 2014, 749). We remember that in the context of quantum measurement Merleau-Ponty said that “[t]he apparatus does not present the object to us” but “realizes a sampling of this phenomenon as well as a fixation. […] Known nature is artificial nature” (Merleau-Ponty 2003, 93). Merleau-Ponty and QBists agree that in quantum mechanics the agent is not an innocent bystander. Instead, measurement is an active, participatory act: “Measurement is not a passive process, but instead a fundamentally *participatory* one” (Fuchs & Stacey 2019, 163).

According to this participatory realism, there is an agent (or a plurality of agents) and an external physical system, and by acting upon the system the agent creates outcomes and these outcomes are the subject matter of quantum mechanics (Fuchs & Stacey 2019, 180). In this sense, “quantum mechanics itself does not deal directly with the objective world; it deals with the experiences of that objective world that belong to whatever particular agent is making use of quantum theory” (Fuchs et al. 2014, 750). Importantly, the agent cannot be reduced to the physical system and the concept of agency is not derivable from quantum mechanics (Fuchs & Stacey 2019, 180).

Even more importantly, this is not to be understood in a Kantian sense such that true reality is hidden behind the phenomena. “In a QBist understanding of quantum theory, it is not that nature is hidden from us. It is that it is not all there yet and never will be; nature is being hammered out as we speak” (Fuchs in Schlosshauer 2011, 285). In this picture, “there is no such thing as *the* universe in any completed and waiting-to-be-discovered sense” (Fuchs in Schlosshauer 2011, 285).

Let us summarize how QBism relates to the scientific realism debate. QBism is *not* realist in the sense that it reifies the mathematical symbols that occur in the formalism of quantum mechanics. Particularly, the wave function is not interpreted as something that exists physically. However, QBism is *anti-instrumentalist* since it holds that there are very important lessons about reality we can learn from quantum mechanics. We learn that we live in a participatory universe and “that reality is *more* than any third-person perspective can capture” (Fuchs 2017, 113). QBism is not solipsist since it presupposes the existence of an external system upon which the agent acts, but it remains unclear how exactly to view the relationship between the agent and physical reality.

There is general consensus that QBism delivers a consistent interpretation of quantum mechanics that avoids problems surrounding the apparent collapse of the wave function and non-locality. However, there seems to be a lack of a clear philosophical foundation. “Now, as a formal proposal, quantum Bayesianism is relatively clear and well developed. But it is rather less transparent philosophically” (Timpson 2008, 580). Perspectivist and phenomenological approaches to science and physics might help QBism to find a suitable philosophical foundation.

What should be clear from this section is that there are many systematically significant parallels between Fuchs’ participatory realism and Merleau-Ponty’s partial realism. Both emphasize the role of the subject (agent) and her experiences and draw attention to the relationship between the observer and the observed (the agent and the external system). Both insist that the physicist is not an innocent bystander, rejecting the idea that scientific observation provides us with an objective picture of nature. Both abstain from mathematizing nature but declare that quantum mechanics quite straightforwardly tells us something very important about the nature and structure of reality. The lessons they draw, of course, are very different from what is claimed by objectivist realists.

One lesson, namely the idea that a purely objective third-person perspective on the world is either impossible or cannot capture all of nature, is at the heart of any perspectivist account. It plays an important role in the phenomenological tradition where we find various degrees of perspectivism. The phenomenological tradition offers a rich experience-first epistemology, developing concepts such as *horizontal intentionality* and *life-world* that could prove immensely useful for advancing perspectivism. The present paper is supposed to pave the way for further attempts to introduce a phenomenological perspectivism into the philosophy of science.

## Conclusion

Perspectivism is a new and promising approach to the scientific realism debate that aims at a middle way between traditional forms of scientific realism and anti-realism. Perspectivism rejects an objectivist picture, according to which the sciences deliver an exhaustive account of nature that is free from all subjective factors. Instead, perspectivism views science as an agent-based endeavor that delivers a certain perspective on nature. This is not to say that the world does not have the features ascribed to it by science, but to deny that there is nothing to know about the world over and above what is described by our best (possible) scientific theories. Although perspectivism is a novel position in current debates, it does have its forerunners. The present paper discusses to which degree perspectivist ideas can be found in the phenomenological tradition. We have seen that Husserl’s account of horizontal intentionality supplements Giere’s analysis of human observation. What is more, Husserl’s claim that all science is epistemically grounded in the life-world and thus can never be entirely free from subjective factors, fits well with Giere’s account of the perspectival character of scientific theories. Then, we have seen how Merleau-Ponty radicalizes this idea by making the further methodological claim that the sciences must incorporate the first-person perspective into science and the ontological claim that reality is in some sense observer-dependent. Importantly, Merleau-Ponty contends that these are lessons that are motivated by science itself, namely by quantum mechanics. In this respect, Merleau-Ponty’s partial realism is in perfect agreement with Fuchs’ participatory realism. In the final section, we indicated how quantum mechanics could support perspectivist and phenomenological approaches and we shed light on QBism which is an interpretation of quantum mechanics particularly close to the ideas we find in Merleau-Ponty.

## Footnotes

1 De Caro lists several contemporary proponents of the view that “only one true and complete description of the world exists, which is typically regarded as being offered by the natural sciences, especially physics” in (De Caro 2020, 58).

2 “But surely, it will be objected, scientists draw conclusions going beyond their instrumentation. Indeed they do. But they do so only by moving to a broader theoretical perspective” (Giere 2006, 49).

3 “My reply is that theoretical claims are also perspectival. The basic idea is that conception is a lot like perception, or, that theorizing is a lot like observing. More specifically, in creating theories, I will argue, scientists create perspectives within which to conceive of aspects of the world” (Giere 2006, 50).

4 “[T]he picture of science that emerges is an agent-based picture” (Giere 2006, 63). Cf. also Giere 2010.

5 We will revisit this distinction between methodological and ontological claims towards the end of Sect. 4.

6 “Like Putnam’s internal realism, perspectivism too is reacting against metaphysical realism and the so-called God’s eye view […] There cannot be an objective, unique, true description of the way the world is as soon as we acknowledge that our scientific knowledge is always from a specific vantage point” (Massimi 2018b, 165).

7 Two new volumes on perspectivim were published in 2020 (Cretu & Massimi 2020; Massimi & McCoy 2020). None of the contributions addresses the phenomenological tradition.

8 For instance, Husserl’s phenomenology had a great influence on Hermann Weyl and the development of his gauge principle (cf. Ryckman 2005; and Ryckman forthcoming).

9 Here, many of Husserl’s insights are in agreement with the findings of early experimental psychologists such as Gestalt psychologists and the members of the Graz school. These ideas have been picked up in the recent movement of experimental phenomenology (cf. Albertazzi 2013). Although neglected for a long time, in the analytic tradition, there have recently been attempts to capture this distinctive character of perceptual experiences (e.g., Church 2013, 50). Particularly notable works in this context that blur the artificial distinction between analytic philosophy and phenomenology are Madary 2017; Smith 2010.

10 A similar remark can be found in Husserl 1973, 116 f. It is interesting to see that the phenomenologically minded mathematician and physicist Hermann Weyl, father of the gauge principle which is one of the cornerstones of modern physics, basically makes the same claim, intentionally using phenomenological terminology (quoted and discussed in Ryckman 2005, 131).

11 Following Jeffrey Barrett (Barrett 1999, 116), Huggett and Wüthrich recently defined “a theory to be *empirically incoherent* in case the truth of the theory undermines our empirical justification for believing it to be true” (Huggett & Wüthrich 2013, 277). Hence, if the life-world is the epistemic foundation of mathematical physics, but mathematical physics is interpreted as revealing that the life-world is a mere illusion, this interpretation of physics is empirically incoherent. Note that this is not to say that our ordinary world of tables and chairs is *ontologically* fundamental. It may well be that physics reveals that our ordinary world is, so to speak, an emergent world, emerging from more fundamental physics such as quantum field theory. For similar discussions in the philosophy of quantum gravity of what it would mean for the very endeavor of physics if it turned out that space and time are not fundamental, cf. Huggett & Wüthrich 2013; Oriti 2014. We must not forget that “[a] central concern of philosophy of science is understanding how the theoretical connects to the empirical, the nature and significance of ‘saving the phenomena’” (Huggett & Wüthrich 2013, 276).

12 “Straightforward experience, in which the life-world is given, is the ultimate foundation of all objective knowledge” (Husserl 1970, 226).

13 “But the researcher of nature does not make clear to himself that the constant fundament of his – after all subjective – work of thought is the surrounding life-world; it is always presupposed as the ground, as the field of work upon which alone his questions, his methods of thought, make sense” (Husserl 1970, 295).

14 Giere states that Jakob von Uexkull’s conception of *Umwelt* “is a more elaborate version of what I am calling a perspective” (Giere 2006, 36). He notes that “this term is borrowed from Husserl, or at least related to Husserl’s use of this term, but I am not in a position to explore this possible connection” (Giere 2006, 123).

15 “Any understanding of reality is by definition perspectival. Effacing our perspective does not bring us any closer to the world. It merely prevents us from understanding anything about the world at all.” (Zahavi 2019, 28)

16 “Phenomenology is not out to dispute the value of science and is not denying that scientific investigations can lead to new insights and expand our understanding of reality. But phenomenologists do reject the idea that natural science can provide an exhaustive account of reality. Importantly, this does not entail that phenomenology is, as such, opposed to quantitative methods and studies. The latter are excellent, but only when addressing quantitative questions.” (Zahavi 2019, 52)

17 A similar approach to Eddington’s example has been championed by Putnam, who explicitly refers to Husserl in this context (Putnam 1987). Cf. De Caro 2020 for affinities between Putnam and perspectivism. Cf. Zahavi 2004 for affinities between Putnam and the phenomenological tradition.

18 One might argue that while the life-world-perspective is *epistemologically* more fundamental than the scientific perspective, the scientific perspective reveals *ontologically* more fundamental aspects in the sense that ordinary objects such as tables and chairs somehow emerge from elementary particles and quantum fields. I see no reason why phenomenologists should deny such a claim.

19 We do know, however, that Husserl was greatly interested in such a project. This is why he supported the work of his pupil Oskar Becker who aimed precisely at a phenomenological grounding of general relativity. In a letter to Weyl, Husserl wrote: “It [Becker’s *Habilitation*] is nothing less than a synthesis of Einstein’s and yours discoveries with my phenomenological investigations on nature. […] What will Einstein say when it is shown that nature requires a relativity-theoretical structure on the a priori grounds of phenomenology and not on positivistic principles, and that only in this way a completely understandable, and ultimately exact, science is possible.” (Husserl in Mancosu & Ryckman 2005, 160 f.)

20 The classical phenomenologists most explicitly arguing that phenomenology and modern physics can in one way or another complement each other are Oskar Becker and Merleau-Ponty. Hermann Weyl and Fritz London are notable figures in early modern physics making similar claims. For phenomenological motifs in Hermann Weyl’s development of the gauge principle, cf. Ryckman 2005 and Ryckman forthcoming; for phenomenological motifs in Fritz London’s interpretation of quantum mechanics, cf. French 2002 and French forthcoming.

21 I am thankful to an anonymous referee of this journal for pressing me on making this clarification.

22 As a side note, it has been argued that general covariance in general relativity (and more precisely gauge invariance in a broader context) “is an indication of the relational character of fundamental observables in physics” (Rovelli 2014, 103). It would be worthwhile to discuss whether general covariance and gauge invariance support perspectivist and phenomenological approaches.

23 This monograph of London and Bauer basically has two objectives: First, to provide a “concise and simple” (London & Bauer 1983, 219) account of the measurement problem in the spirit of von Neumann’s groundbreaking *Mathematische Grundlagen der Quantenmechanik* (1932). Providing the axiomatic foundations of quantum mechanics, von Neumann’s book was one of the most influential works of early quantum mechanics. London and Bauer were in broad agreement with von Neumann. They did not understand their monograph as a counter project but as a more accessible version of von Neumann’s highly technical work which was written in German. Secondly, London and Bauer shed more light on the relationship between the observed and the observer, aiming at clarifying the role of consciousness in quantum measurement. In perfect agreement with Merleau-Ponty they hold that modern physics reveals that “the idea of an observable world totally independent of the observer, was a vacuous idea” (London & Bauer 1983, 220). For an analysis of the phenomenological motifs in Fritz London’s approach to quantum mechanics and for how this approach can serve as a starting point for a genuinely phenomenological interpretation of quantum mechanics, cf. French 2002 and particularly French forthcoming.

24 The quoted article by Adam Frank, Marcelo Gleiser, and Evan Thompson is a great example: https://aeon.co/essays/the-blind-spot-of-science-is-the-neglect-of-lived-experience?fbclid=IwAR1QpuiKEPuaE3aH37xTZA6bhHs9vstxBXt_Znw84NFkSEBdj389BuAirsA. Retrieved on February 13, 2020.

25 For a discussion of unobservable scientific entities from a phenomenological perspective, cf. Wiltsche 2012; Berghofer 2018b.

26 . Retrieved on February 13, 2020. In this context, cf., e.g., Karaca 2017, 344.

27 For discussions of what kind of observation is taking place in LHC experiments, cf., e.g., Beauchemin 2017; Karaca 2017, 2018. Concerning the relationship between theory and observation, Beauchemin argues that reflection on such big data experiments “indicates that the frontier between theory and observation is blurry and that the dichotomy theory-experiment should be revised” (Beauchemin 2017, 275). Although Beauchemin stresses the theory-ladenness of LHC experiments, he does not dispute the objectivity of the corresponding empirical facts. Similarly, Karaca argues that “the exploratory data selection procedure carried out in the ATLAS experiment is *theory-laden* in the sense that its implementation is crucially dependent on the aforementioned theoretical models that the experiment is aimed to test” (Karaca 2017, 350; cf. also Karaca 2018, 5449), insisting, however, that *no* vicious circularity is happening there (Karaca 2018, 5450). Werner Callebaut has explicitly argued that Giere’s perspectivism “provides the best resources currently at our disposal to tackle many of the philosophical issues” surrounding big data *biology* (Callebaut 2012, 69).

28 Unfortunately, in the English translation (Merleau-Ponty 2003, 98), the quotation marks are missing.

29 Merleau-Ponty’s partial realism denies the existence of individual observer-independent physical objects and intrinsic properties. Instead, as we have seen, it regards the “structural relations” that refer “to certain mathematical forms necessary for the description of the relation of the subject to the object” as the fundamental entities. Accordingly, Merleau-Ponty’s partial realism has much common ground with structural realism. In current debates, structural realism is a form of selective realism that focuses on the mathematical structure of scientific theories. A distinction is made between epistemic and ontic structural realism. Broadly speaking, while epistemic structural realism says that structures are all we can *know*, ontic structural realism (OSR) says that structures are all there *is*. OSR enjoys much popularity and comes in many flavors, ranging from the claim that objects (at the fundamental level) do not possess intrinsic properties to the eliminativist version, according to which there are no objects but only structures (for more details, cf. Ladyman 2016; Berghofer 2018c). Of course, the difference between Merleau-Ponty and proponents of OSR is that the latter do not regard their structural relations as relations between subject and object. Proponents of OSR presuppose the observer-independence of physical reality.

30 However, it should be mentioned that there are some passages in Husserl’s oeuvre that suggest that physics in its most elaborated form would successfully make the physicist and her life-world subjects of investigation, abandoning the idea of a purely objective third-person perspective (Husserl 1970, 295; 2002, 287). It is not entirely clear, however, whether here Husserl wants to say that physics must be phenomenologically clarified so that physics (as it is) can be ultimately grounded and justified. Or whether his claim is to be understood in the Merleau-Pontyan sense that physics can only succeed in its goal of clarifying nature if it succeeds in incorporating the physicist *into the physical theories*.

31 “There is no pure third-person perspective, just as there is no view from nowhere. This is, of course, not to say that there is no third-person perspective, but merely that such a perspective is, precisely, a perspective from somewhere. It is a view that *we* can adopt on the world.” (Zahavi 2019, 54)

32 Cf. the beginning of section 2 for a similar distinction we made in the context of perspectivism.

33 However, it should be mentioned that even in deterministic Bohmian mechanics, “there are unavoidable limitations to our knowledge of particles. In fact, once the wave function is prepared, there is an *absolute uncertainty* regarding the positions of the particles” (Solé et al. 2016, 22).

34 Similarly, QBism dissolves another problem that has been puzzling physicists, namely non-locality (cf. Fuchs et al. 2014; Timpson 2008).

35 This objection has been raised, e.g., by Chen (2019, 6). For how the objection of empirical incoherence emerges from phenomenological reasoning, cf. the beginning of Sect. 2 of the present paper, particularly footnote 11.

## References

[CR1] Albert D, Cushing J, Fine A, Goldstein S (1996). Elementary quantum metaphysics. Bohmian mechanics and quantum theory: An appraisal.

[CR2] Albertazzi L (2013). Handbook of experimental phenomenology.

[CR3] Barrett J (1999). The quantum mechanics of minds and worlds.

[CR4] Beauchemin P-H (2017). Autopsy of measurements with the ATLAS detector at the LHC. Synthese.

[CR5] Berghofer P (2018). Husserl’s conception of experiential justification: What it is and why it matters. Husserl Studies.

[CR6] Berghofer P (2018). Transcendental phenomenology and unobservable entities. Perspectives.

[CR7] Berghofer P (2018). Ontic structural realism and quantum field theory: Are there intrinsic properties at the most fundamental level of reality?. Studies in History and Philosophy of Modern Physics.

[CR8] Berghofer P (2020). Towards a phenomenological conception of experiential justification. Synthese.

[CR9] Bitbol, M. (forthcoming). A phenomenological ontology for physics: Merleau-Ponty and QBism, forthcoming. In P. Berghofer & H. Wiltsche (Ed), *Phenomenological approaches to physics*. Synthese Library, Springer.

[CR10] Brown M (2009). Models and perspectives on stage: Remarks on Giere’s scientific perspectivism. Studies in History and Philosophy of Science.

[CR11] Bub J (2004). Why the quantum?. Studies in History and Philosophy of Modern Physics.

[CR12] Callebaut W (2012). Scientific perspectivism: A philosopher of science’s response to the challenge of big data biology. Studies in History and Philosophy of Biological and Biomedical Sciences.

[CR13] Carroll S, Singh A, Aguirre A, Foster B, Merali Z (2019). Mad-dog everettianism: Quantum mechanics at its most minimal. What is fundamental?.

[CR14] Caves C, Fuchs C, Schack R (2002). Quantum probabilities as Bayesian probabilities. Physical Review A.

[CR15] Chakravartty A (2010). Perspectivism, inconsistent models, and contrastive explanation. Studies in History and Philosophy of Science.

[CR16] Chakravartty, Anjan (2017). Scientific realism. Edward N. Zalta (ed.), *The Stanford encyclopedia of philosophy* (summer 2017 edition). URL = <https://plato.stanford.edu/archives/sum2017/entries/scientific-realism/>.

[CR17] Chakravartty A, Saatsi J (2018). Realism, antirealism, epistemic stances, and voluntarism. The Routledge handbook of scientific realism.

[CR18] Chen, E. K. (2019) Realism about the wave function. *Philosophy Compass, 14*(7). 10.1111/phc3.12611.

[CR19] Chiribella G, D’Ariano G, Periotti P (2011). Informational derivation of quantum theory. Physical Review A.

[CR20] Church J (2013). Possibilities of perception.

[CR21] Cretu A-M, Massimi M (2020). Knowledge from a human point of view.

[CR22] De Caro M, Cretu A-M, Massimi M (2020). Hilary Putnam on perspectivism and naturalism. Knowledge from a human point of view.

[CR23] Dieks D (2019). Quantum reality, perspectivalism and covariance. Foundations of Physics.

[CR24] Dieks D, Lombardi O, Fortin S, López C, Holik F (2019). Quantum mechanics and perspectivalism. Quantum worlds: Perspectives on the ontology of quantum mechanics.

[CR25] Eddington A (1928). The nature of the physical world.

[CR26] Fine A, Leplin J (1984). The natural ontological attitude. Scientific realism.

[CR27] Forbes C (2017). A pragmatic, existentialist approach to the scientific realism debate. Synthese.

[CR28] French S (2002). A phenomenological solution to the measurement problem? Husserl and the foundations of quantum mechanics. Studies in the History and Philosophy of Modern Physics.

[CR29] French, Steven (forthcoming). From a lost history to a new future: Is a phenomenological approach to quantum physics viable? forthcoming in P. Berghofer & H. Wiltsche, *Phenomenological approaches to physics*. Synthese Library, Springer.

[CR30] Frescura, F. A. M. & Hiley, Basil (1984). Algebras, quantum theory and pre-space. *Revista Brasileira de Fisica*, Volume Especial, Julho 1984, Os 70 anos de Mario Schonberg, 49–86.

[CR31] Frost-Arnold G (2010). The no-miracles argument for realism: Inference to an unacceptable explanation. Philosophy of Science.

[CR32] Fuchs, C. (2017). On participatory realism. In I. Durham & D. Rickles (Eds.), *Information and interaction* (pp. 113–134). Cham: Springer.

[CR33] Fuchs C, Mermin D, Schack R (2014). An introduction to QBism with an application to the locality of quantum mechanics. American Journal of Physics.

[CR34] Fuchs C, Schack R (2015). QBism and the Greeks: Why a quantum state does not represent an element of physical reality. Physica Scripta.

[CR35] Fuchs C, Stacey B, Chiribella G, Spekkens R (2016). Some negative remarks on operational approaches to quantum theory. Quantum theory: Informational foundations and foils.

[CR36] Fuchs C, Stacey B, Rasel E, Schleich W, Wölk S (2019). QBism: Quantum theory as a hero’s handbook. Foundations of quantum theory.

[CR37] Giere R (2006). Scientific perspectivism.

[CR38] Giere R (2010). An agent-based conception of models and scientific representation. Synthese.

[CR39] Giere R (2013). Kuhn as perspectival realist. Topoi.

[CR40] Giere R (2016). Feyerabend’s perspectivism. Studies in History and Philosophy of Science.

[CR41] Goyal P (2012). Information physics – towards a new conception of physical reality. Information.

[CR42] Griffiths D (2018). Introduction to quantum mechanics.

[CR43] Hartimo, M. (2018). Husserl’s scientific context 1917–1938, a look into Husserl’s private library. *The New Yearbook for Phenomenology and Phenomenological Philosophy, 16*, 335–355.

[CR44] Healey R (2012). Quantum theory: A pragmatist approach. British Journal for the Philosophy of Science.

[CR45] Heisenberg W, Price W, Chissick S (1977). Remarks on the origin of the relations of uncertainty. The uncertainty principle and foundations of quantum mechanics.

[CR46] Heisenberg W, Wheeler JA, Zurek WH (1983). The Physical Content of Quantum Kinematics and Mechanics. Quantum theory and measurement.

[CR47] Hilgevoord, Jan & Uffink, Jos (2016). The uncertainty principle. In Edward N. Zalta (ed.), *The Stanford encyclopedia of philosophy* (winter 2016 edition), URL = <https://plato.stanford.edu/archives/win2016/entries/qt-uncertainty/>.

[CR48] Huggett, N., & Wüthrich, C. (2013). Emergent spacetime and empirical (in)coherence. *Studies in History and Philosophy of Modern Physics, 44*, 276–285.

[CR49] Husserl E (1960). Cartesian meditations.

[CR50] Husserl E (1969). Formal and transcendental logic.

[CR51] Husserl E (1970). The crisis of European sciences and transcendental phenomenology.

[CR52] Husserl E (1972). Erfahrung und Urteil.

[CR53] Husserl E (1973). Zur Phänomenologie der Intersubjektivität. Texte aus dem Nachlass. Erster Teil. Husserliana XIII.

[CR54] Husserl E (1977). Phenomenological psychology.

[CR55] Husserl E (1982). Ideas pertaining to a pure phenomenology and to a phenomenological philosophy, first book.

[CR56] Husserl E (1984). Einleitung in die Logik und Erkenntnistheorie, Vorlesungen 1906/1907.

[CR57] Husserl E (2001). Analyses concerning passive and active synthesis.

[CR58] Husserl E (2002). Logische Untersuchungen, Ergänzungsband, Erster Teil: Entwürfe zur Umarbeitung der VI. Untersuchung und zur Vorrede für die Neuauflage der Logischen Untersuchungen (Sommer 1913).

[CR59] Karaca K (2017). A case study in experimental exploration: Exploratory data selection at the large hadron collider. Synthese.

[CR60] Karaca K (2018). Lessons from the large hadron collider for model-based experimentation: The concept of a model of data acquisition and the scope of the hierarchy of models. Synthese.

[CR61] Ladyman, James (2016). Structural realism. Edward N. Zalta (ed.), *The Stanford encyclopedia of philosophy* (winter 2016 edition). URL = <https://plato.stanford.edu/archives/win2016/entries/structural-realism/>.

[CR62] Landy D, Goldstone R (2007). How abstract is symbolic thought?. Journal of Experimental Psychology.

[CR63] London F, Bauer E, Wheeler JA, Zurek WH (1983). The theory of observation in quantum mechanics. Quantum theory and measurement.

[CR64] Madary M (2017). Visual phenomenology.

[CR65] Mancosu P, Ryckman T, Peckhaus V (2005). Geometry, physics and phenomenology: Four letters of O. Becker to H. Weyl. Oskar Becker und die Philosophie der Mathematik.

[CR66] Massimi M (2012). Scientific perspectivism and its foes. Philosophia.

[CR67] Massimi M (2018). Four kinds of perspectival truth. Philosophy and Phenomenological Research.

[CR68] Massimi M, Saatsi J (2018). Perspectivism. The Routledge handbook of scientific realism.

[CR69] Massimi M, McCoy C (2020). Understanding perspectivism.

[CR70] Merleau-Ponty M (1968). The visible and the invisible.

[CR71] Merleau-Ponty, M. (2003). Nature: Course notes from the Collège de France. Evanston: Northern University Press.

[CR72] Merleau-Ponty M (2004). The world of perception.

[CR73] Merleau-Ponty M (2012). Phenomenology of perception.

[CR74] Mermin D (2014). QBism puts the scientist back into science. Nature.

[CR75] Oriti D (2014). Disappearance and emergence of space and time in quantum gravity. Studies in History of Philosophy of Modern Physics.

[CR76] Rovelli C (1996). Relational quantum mechanics. International Journal of Theoretical Physics.

[CR77] Rovelli C (2014). Why gauge?. Foundations of Physics.

[CR78] Ryckman T (2005). The reign of relativity: Philosophy of physics 1915–1925.

[CR79] Ryckam, Thomas (forthcoming). The gauge principle, Hermann Weyl, and symbolic construction from the ‘Purely Infinitesimal’” forthcoming in P. Berghofer & H. Wiltsche. *Phenomenological approaches to physics*. Synthese Library, Springer.

[CR80] Schlosshauer M (2011). Elegance and enigma: The quantum interviews.

[CR81] Sellars W (1963). Science, perception and reality.

[CR82] Smith J (2010). Seeing other people. Philosophy and Phenomenological Research.

[CR83] Solé A, Oriols X, Marian D, Zanghi N (2016). How does quantum uncertainty emerge from deterministic Bohmian mechanics?. Fluctuation and Noise Letters.

[CR84] Stein E (2004). Einführung in die Philosophie.

[CR85] Teller P (2001). Twilight of the perfect model model. Erkenntnis.

[CR86] Teller P (2011). Two models of truth. Analysis.

[CR87] Teller P, Massimi M, McCoy C (2020). What is perspectivism, and does it count as realism?. Understanding perspectivism.

[CR88] Timpson C (2008). Quantum Bayesianism: A study. Studies in History and Philosophy of Modern Physics.

[CR89] de La Tremblaye, L. (forthcoming). QBism from a phenomenological point of view: Husserl and QBism, forthcoming in P. Berghofer & H. Wiltsche. *Phenomenological approaches to physics*. Synthese Library, Springer.

[CR90] Vaidman L, Gao S (2014). Protective measurement of the wave function of a single system. Protective measurement and quantum reality.

[CR91] Wallace D, Ney A, Albert D (2013). A prolegomenon to the ontology of the Everett interpretation. The wave function.

[CR92] Wiltsche H (2012). What is wrong with Husserl’s scientific anti-realism?. Inquiry.

[CR93] Zahavi D, Carr D, Chan-Fai C (2004). Natural realism, anti-reductionism, and intentionality. The ‘phenomenology’ of Hilary Putnam. Space, time, and culture.

[CR94] Zahavi D (2019). Phenomenology: The basics.

